# New interpretation of the Gran Dolina-TD6 bearing *Homo antecessor* deposits through sedimentological analysis

**DOI:** 10.1038/srep34799

**Published:** 2016-10-07

**Authors:** I. Campaña, A. Pérez-González, A. Benito-Calvo, J. Rosell, R. Blasco, J. M. Bermúdez de Castro, E. Carbonell, J. L. Arsuaga

**Affiliations:** 1 Centro Nacional de Investigación sobre la Evolución Humana (CENIEH), Paseo de la Sierra de Atapuerca 3, 09002 Burgos, Spain; 2 Grupo Espeleológico Edelweiss, Paseo del Espolón s/n, 09071 Burgos, Spain; 3 IPHES, Institut Català de Paleoecología Humana i Evolució Social. C/ Marcelli Domingo s/n Campus Sescelades URV (Edifici W3). 43007 Tarragona,Spain; 4 Universitat Rovira i Virgili (URV), Campus Catalunya, Avinguda de Catalunya 35, 43002 Tarragona, Spain; 5 Department of Anthropology, University College of London, 14 Taviton Street, London WC1H 0BW, UK; 6 Centro de Investigación Sobre la Evolución y Comportamiento Humanos, Universidad Complutense de Madrid-Instituto de Salud Carlos III, Madrid, Spain; 7 Departamento de Paleontología, Facultad de Ciencias Geológicas, Universidad Complutense de Madrid, Madrid, Spain

## Abstract

Gran Dolina is a cavity infilled by at least 25 m of Pleistocene sediments. This sequence contains the TD6 stratigraphic unit, whose records include around 170 hominin bones that have allowed the definition of a new species, *Homo antecessor.* This fossil accumulation was studied as a single assemblage and interpreted as a succession of several human home bases. We propose a complete stratigraphic context and sedimentological interpretation for TD6, analyzing the relationships between the sedimentary facies, the clasts and archaeo-palaeontological remains. The TD6 unit has been divided into three sub-units and 13 layers. Nine sedimentary facies have been defined. Hominin remains appear related to three different sedimentary facies: debris flow facies, channel facies and floodplain facies. They show three kinds of distribution: first a group of scattered fossils, then a group with layers of fossils in fluvial facies, and third a group with a layer of fossils in mixed fluvial and gravity flow facies. The results of this work suggest that some of these hominin remains accumulated in the cave by geological processes, coming from the adjacent slope above the cave or the cave entry, as the palaeogeography and sedimentary characteristics of these allochthonous facies suggest.

TD6 is surely the most well-known lithostratigraphic unit in the Gran Dolina site. Human and non-human remains there have been the main focus of a number of studies^1^,^2^,^3^,^4^,^5^,^6^,^7^,^8^, which have enriched our knowledge about the human lineage during the Early Pleistocene and the palaeoecological context. In 1995, *H. antecessor* was the oldest known hominin taxa in Western Europe, and its discovery changed the paradigm about the first hominin settlement in Europe^9^.

TD6 is situated in the centre of the stratigraphic section of the Gran Dolina site (Sierra de Atapuerca, Burgos, Spain) (Fig. 1). The opening of the Gran Dolina cavity to the outside during the Early Pleistocene^10^ resulted in allochthonous sediment input and the accumulation of archaeo-palaeoanthropological remains. These sediments have been differentiated into 12 lithostratigraphic units^11^,^12^. The sediment source area for Gran Dolina corresponds to the hillslope of the southwest flank of the Sierra de Atapuerca, where several phases of low rate fluvial incision and slope retreat related to infilling of the trench sites have been described^13^.

TD6 stratigraphy and chronology have been extensively studied by different authors^14^,^15^,^16^,^17^,^18^,^19^,^20^,^21^,^22^,^23^,^24^,^25^,^26^,^27^,^28^. In these studies, a reversal of magnetostratigraphic polarity in TD7 was reported, attributed to the Matuyama-Brunhes boundary^26^,^27^,^28^. Recently, Campaña *et al*.^29^ described the main sedimentary facies of the Gran Dolina site, including the TD6 sedimentary facies.

Up to the present time, TD6 has been excavated in two phases. The first excavation was during the 1994–1995 field seasons, in a test pit of approximately 6 m^2^, where several hominin remains and lithic artefacts were recovered from a distinctive layer named the “Aurora Stratum”^1^,^26^. The test pit was situated in the south of the Gran Dolina site (Fig. 1) and its south-east area was affected by cut-and-fill, with loss of information over about 1 meter diameter of the test pit. The second excavation began in the first decade of the present century. This excavation area had a triangular shape of 13 m^2^, and was situated in the central area of the Gran Dolina section (Fig. 1), where medial sedimentary facies appear. The northern section of TD6 (namely “Torreón”) was excavated during these years and hominin fossils were also found there.

About 170 fossils of *H. antecessor* were found in TD6.2, as well as more than 800 lithic artefacts made of flint, quartzite, sandstone, limestone and quartz, classified as Mode 1 technology^1^. In addition, more than 6500 mammal fossils were recovered from all TD6 units. *H. antecessor* is characterized by a combination of primitive traits shared with early *Homo*, primitive traits retained by modern humans, and derived traits also shared with modern humans^2^,^3^,^27^. According to these characteristics, *H. antecessor* seems to represent a European lineage which is different from other African and Asian lineages^28^. The particular combination of features found in *H. antecessor* allows to assume that this species is close to the last common ancestor of Neanderthals and modern humans^30^.

Although several studies had been carried out on the fossil and lithic remains after the second excavation^30^, our knowledge of the sedimentology and stratigraphy of TD6 is still incomplete. The aim of this paper is to propose a detailed sedimentological and stratigraphical interpretation for the TD6 stratigraphic unit, extending previous^29^, and to study their relationships with the spatial distribution and sorting of the archaeo-palaeontological remains.

## Results

### TD6 sedimentary facies

TD6 has been separated into sedimentary facies based on field observations, particle size analyses and recent cave sediment classifications^31^,^32^ (Table 1, Figs 2 and 3, SI 1). In this work, we extended previous classifications^12^,^25^,^28^,^29^, focusing on the stratigraphic layers and sedimentary environments containing archaeological remains. The latter are mainly associated with proximal debris flow facies (Facies D1) and floodplain facies including boulders (Facies F).

### Facies D1: Matrix-supported debris flow

This facies is characterized by a mixture of chaotic and unsorted sediments from medium sized boulders to gravels, sands and mud. Facies D1 is mainly composed of matrix-supported boulders and gravels with a muddy matrix, including local clast-supported areas. The clasts in this facies represent 30–35% of the area measured in the stratigraphic section. The matrix is mainly composed of about 75% of clayey silt with about 25% of gravel. The amount and size of boulders decreased towards the south-east, towards the dip direction of this facies. These characteristics indicate that the entrance of this facies was positioned in the north-west. Facies D1 is found in TD6.2 and TD6.3. The characteristics of this facies coincide with the diamicton facies described in caves, and interpreted as debris flow processes^33^,^34^.

### Facies D2: Aligned-clasts debris flow

Facies D2 is formed by clast-supported small boulders with a gravel and muddy matrix. It is characterized by aligned clasts that indicate the entrance direction and could suggest a unique punctual sediment input for its formation. This facies occurs in TD6.1, with a constant thickness of about 0.3 m. The layer containing this facies dips towards the south-east, suggesting a north-west location of the entrance. Boulders are sub-angular and tabular limestone ranging from 10 to 30 cm in size, and they represent about 40% of the section area. The matrix has up to 25% gravel.

### Facies F: Floodplain and debris flow

Facies F is composed of silts and clays with small boulders and gravel. It is similar to floodplain facies (Facies E), showing the same matrix particle size distribution and the same massive structure. As with Facies E (Tables 1, SI 1), Facies F is associated with channel, but Facies F is only found in TD6.2 (Fig. 3). Because of these traits, we interpret Facies F as also being floodplain facies. Nevertheless, this facies includes several limestone clasts, which are medium to very small sub-angular boulders that represent about 20% of the section. These boulders appear dispersed in the layers as matrix-supported, unsorted, structureless and with no preferred orientation. The presence of these clasts does not agree with a floodplain environment as observed in Facies E, and seems to indicate a secondary sedimentological process in the development of Facies F. The amount, size and shape of the limestone boulders suggest a gravity flow process and the dip of the layers towards the north-west (Fig. 4) indicates a southern entry, although this dip is exaggerated by the deformation process. Facies F is explained as the result of two geological processes: floodplain and debris flow. Because of the similar matrix characteristics produced by these two processes, the lateral change separating both environments could not be mapped.

### TD6 Stratigraphic sequence

**Figure 1:**
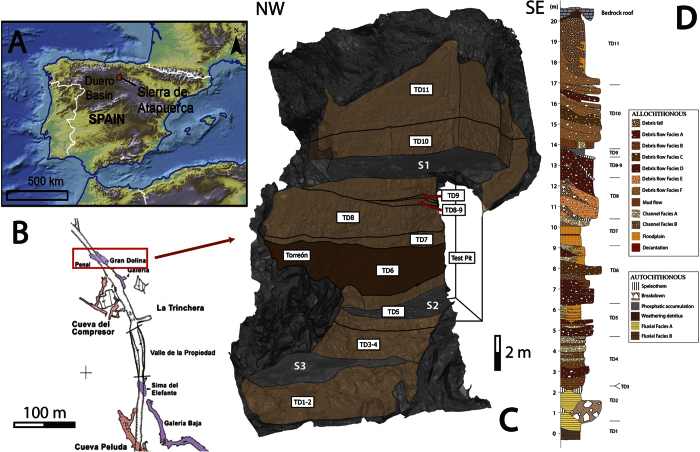
(**A**) Location of the Sierra de Atapuerca. (**B**) Location of Gran Dolina site situated in a railway trench (“Trinchera”)^58^. (**C**) 3D model of the Gran Dolina site in 2012. Brown areas indicate stratigraphic units. Grey areas are the wall and roof of the cave. The excavation surfaces in 2012 have been marked with the letter S. S1 – TD10 excavation surface. S2 – TD5 excavation surface. S3 – TD4 excavation surface. The site of “Torreón” and the test pit excavation are situated in TD6. (**D**) Stratigraphic units (TD1 to TD11) and sedimentary facies of the Gran Dolina site 19. 1A map created by ArcGis 10.1 using the elevation data of free access dataset SRTM90 (http://www.cgiar-csi.org/data/srtm-90m-digital-elevation-database-v4-1). 3D model of 1C created by 3DReshaper 8.1 software (http://www.3dreshaper.com/en/).

**Figure 2:**
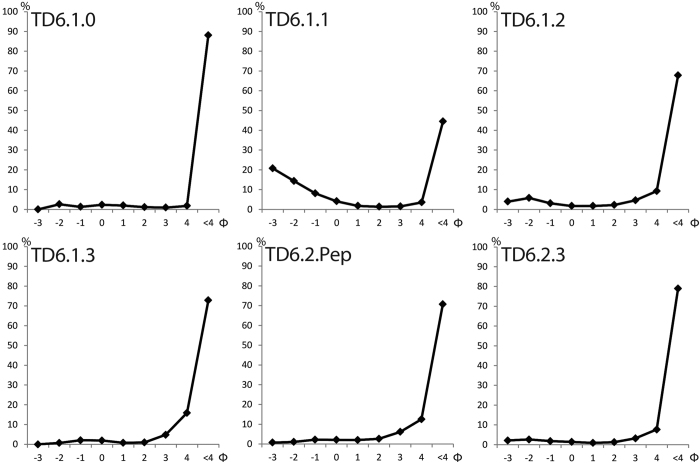
Particle size distribution of six layers of TD6.1 and TD6.2 sub-units in the Gran Dolina site. Abscissa shows Phi size. Ordinate shows percentage.

TD6 is a 3 m thick lithostratigraphic unit situated in the middle of the 25 m sedimentary infill of the Gran Dolina site. It is bounded by two sedimentary discontinuities. The lower discontinuity is indicated by an important sedimentary change where the TD5.1 predominance of sorted gravels and mud (similar to Facies A and Facies E) changes to unsorted clasts in a muddy matrix (Facies D1). The upper discontinuity is a sedimentological change from red mud with hyena coprolites (Facies G) to TD7 laminated silts (Figs 1 and 5). TD6 is divided into three sub-units^12^,^28^: TD6.3, TD6.2 and TD6.1 from bottom to top (Fig. 6). In this study, these sub-units have also been divided into various layers according to the facies changes and identified discontinuities (Table 2, Fig. 5).

Geochemical analysis carried out on the different layers and sedimentary facies showed similar compositional results due to the identical source area and the short transport distances (Supplementary Information 2).

### Analyses of clasts and palaeo-archaeological remains in TD6.1 and TD6.2

The means and standard deviation values calculated for the length of clasts, fossils and lithic tools are displayed by layers and facies in Table 3.

The archaeological remains show a clear sorting by sedimentary facies, as seen in Table 3. The higher energy facies not only have larger remains, but more fossils and lithic tools were also found in these facies. The higher size of clasts with respect to fossils and lithic tools was expected due to the fact that only clasts longer than 100 mm were measured in the excavation. Facies E of TD6.1 is not shown because insufficient remains were found in this layer. Mean and standard deviation results indicate a significantly higher clast size in Facies D1 than in the other facies (Table 3). This is expected in debris flow facies, as it has more energy than fluvial facies. This pattern is also observed in the size of the fossil remains, where debris flow facies (D1 and D2) have higher fossil sizes (>40 mm) than the other facies. However, it is noticeable that Facies F has fossil size values similar to debris flow facies. Lithic tool sizes show different results (Table 3), where floodplain facies (E and F) have higher values. However, we have to be cautious with Facies E results, since the sample here only contains seven lithic tools.

### Spatial distribution of the palaeo-archaeological remains in the test pit

The spatial distributions of clasts, lithic tools, mammal and human fossils are shown in Fig. 7. Two IDW (inverse distance weighting) surfaces have been generated for each group using the length of the items and the Z coordinate of the remains. These analyses have been done over the remains of the test pit. These remains belong to a condensed layer that comprises of different layers^35^ of sedimentary facies F. In these spatial distributions we are surely observed the remains of TD6.2.2, TD6.2.3 and TD6.2.4.

The eight IDW surfaces generated show that the spatial distributions observed are similar in the four groups (Fig. 8). In all instances, the greater accumulations of remains are observed in the south-west and the existence of an empty circular area was seen indicating the position of the cut-and-fill.

The distribution of fossil size does not show any significant pattern. The size of hominin remains is larger in the south-west and the IDW surface suggests that the length size of hominin remains is larger than for fossil remains. Although this could be due to larger sizes of hominin fossils, in fact, the low amount of hominin fossils means that the few hominin fossils greater than 90 mm are overrated in the interpolation, and the results show areas with hominin fossils greater than 90 mm. This is easily observable in the fact that area above 90 mm in the IDW surface of hominin fossils only has one point, that is, it has been generated from a single fossil. The lithic tools also show no size pattern.

The IDW surfaces generated with the Z coordinates show the same strike and dip of the layers for the four groups of remains. The four surfaces mainly have a westerly dip with the lowest area situated in the east (Fig. 8).

## Discussion

### Sedimentary facies

**Figure 3:**
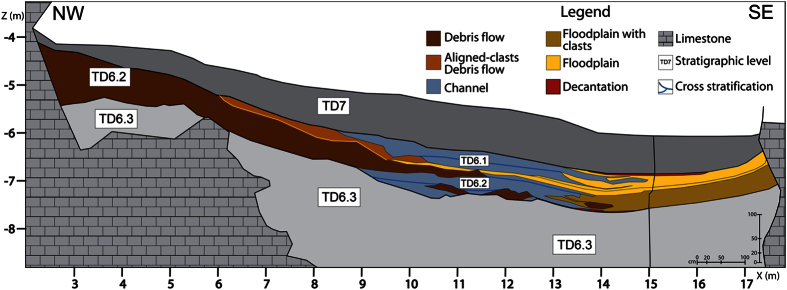
Sedimentary environment facies distribution and stratigraphic section of TD6.1 and TD6.2 sub-units in the Gran Dolina site.

The nine facies distinguished show that TD6 is mainly a clastic unit formed by sediment gravity flows and fluvial flows (Fig. 3), whose geochemical composition does not show significant variations (Supplementary Information 2). Other data, such as fossil or pollen analysis, were collected considering only the sub-units of TD6, since detailed stratigraphic classifications in the test pit were not available at that time.

TD6.3 is mainly a sediment gravity flow sub-unit where channel flows are restricted and poorly developed (Facies B). Pollen data postulate a drier climate at the base of TD6^36^, although microfossil studies suggest the opposite^37^,^38^. Facies D1, described in the lowest sub-layer of TD6 (TD6.3.3.4, Table 2), is anomalous because it includes more very small clast-supported boulders than other examples of Facies D1. This characteristic could be related to sedimentological reactivation in the cave after a stable period.

The next sub-layers are formed by Facies B, D1 and H. This is interpreted as sediment gravity flow inputs to the cave from a north-west direction and minor fluvial flows circulating over them. Features of Facies B indicate that this sediment deposit was formed by a small ephemeral flow with poorly sorted grain size that moved over the debris flow, similar to a braided river^39^.

TD6.3.3.1 is a mud layer interpreted as Facies G, with the same 5 cm thickness in all sections (Fig. 5). This decantation layer could indicate a flooding event from the south of the cavity and slow sedimentation rates or a sub-aqueous debris flow input^32^,^40^. TD6.3.2 continued with sediment gravity flow events and finished with Facies B, thicker than in the lower layers. This fluvial flow (TD6.3.2.1) could be the beginning of the stream flow that is observed in TD6.2 and TD6.1, interrupted by the input of Facies C of TD6.3.1. In this case, mud layers situated in the south could be considered to be floodplain facies (Facies E), and not mud flow facies (Facies H). But these mud layers do not show the different characteristics of previous layers that are undoubtedly Facies H, supporting by micromorphological study that indicate a sediment gravity flow as environment^25^. Facies C of TD6.3.1 is formed by large clasts and is situated in the centre of the stratigraphic section (Fig. 5). This new position could denote another entry direction, perpendicular to the actual stratigraphic section, or the existence of palaeorelief which controlled the sedimentation. Facies C has larger clasts than in lower layers which suggest higher energy in the sediment gravity flow.

**Figure 4:**
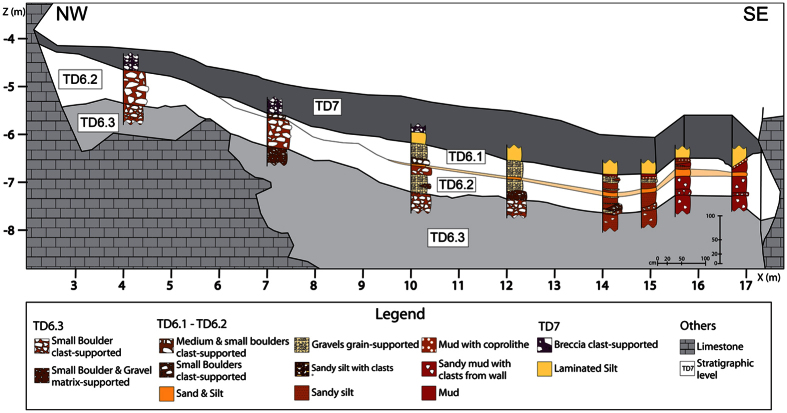
Lithofacies distribution of the TD6.1 and TD6.2 sub-units in Gran Dolina site.

The first input of TD6.2 is a gravel deposit in the centre of the section (Figs 3 and 6) indicating a channel facies. The first gravitational input is TD6.2.4-Jordi. This layer dips to the north-west (Fig. 4), the opposite direction from lower and upper units of TD6. This dip is also observed in the spatial distribution of the archaeological remains and clasts (Fig. 8), where a mainly westerly dip is seen. This current dip is the consequence of a post-depositional deformation caused by subsidence in this area of the section. The layer is composed of Facies F which is described as a floodplain and debris flow deposit. Floodplain environments are low energy flows with little capability to drag gravels or boulders. In addition, the distribution of limestone clasts, their mean size and standard deviations are similar to sedimentary Facies D1 and D2 (Table 3), interpreted as debris flow deposits. Micromorphological studies indicate that the depositional setting for this layer was an aqueous environment of high and medium energy^25^. These data support the interpretation of this Facies as a mixture of sedimentological processes. Therefore, during the deposition of this layer, a secondary entry from the south was active. The inputs from this entry mixed with the floodplain sediments.

Two clayey mud layers are found at the bottom and on the top of TD6.2.4-Jordi (Fig. 4). These layers are a few centimetres thick in the section, but their thicknesses increase up to 30 cm towards the excavated area, where the lowest local topography should be situated. These layers represent a cessation of stream flow in this area, characterized by low sedimentation rates and energy^25^,^32^,^40^, they are related to the Facies A from the centre of the section and can be assigned to Facies G.

TD6.2.3 and TD6.2.2 are Facies A (channel facies) and Facies F (floodplain and debris flow facies) that indicate the formation of a fluvial environment inside the cave. This interpretation is supported by micromorphological data, which suggested an aqueous environment for these layers^25^. The formation of this fluvial environment suggests a relative increase in humidity in TD6.2, which is in agreement with the palaeoenvironmental reconstruction using herpetofauna^41^,^42^. Considering the mean and standard deviation of the clasts, fossil and lithic remains, Facies A has lower size values with respect to Facies D1, D2 and F (Table 3). This suggests a different archaeological content pattern according to the sedimentological environment, as expected in a well-sorted layer, perhaps suggesting that these remains could be brought by the fluvial flow. The size of the biggest clasts in the channel facies indicates the maximum size that the flow could carry. This means that the flow could drag larger fossil remains than the fossil mean that we observe. The could be due to the lack of bones with larger sizes, but the presence of fossils with higher sizes in other facies (Table 3) negates this idea. In fact, the largest size of fossil remains is 170 mm, which is greater than the mean size of the clasts. Though these clasts were only in the *lag* layers indicating that, although sometimes the flow was able to drag boulders (>64 mm), in general, its energy was lower and less capable to drag larger sizes. Although a certain source of wall rock could exist overestimating the size mean. Otherwise, the amount and size of clasts in Facies F suggest that the secondary entry of TD6.2.4-Jordi was still active. The separation between TD6.2.3 and TD6.2.2 is due to a *lag* layer identified in the base of each layer.

Some clasts in Facies F show weathering crusts on their surfaces. These crusts have been identified as phosphate minerals, mainly hydroxyl-apatite, and indicate a lower pH environment. The alteration is only located in the south-east, in the test pit, and is only observed on the limestone clasts. The source of the phosphate was surely from the TD6.1.0 layer, where hyena coprolites were found.

The main environment to the north corresponds with sediment gravity flows (Facies D1). The shape of this deposit denotes that it was input from the north-west, in the place called “Torreón” (Figs 1 and 9). The mean size and standard deviations of the limestone clasts (Table 3), particle size distribution and unsorted sediments indicate high-density flows which dragged the sediment from the outside. In “Torreón”, the layer has its maximum thickness and some clasts have a sub-vertical dip (about 10%), which suggests a sub-vertical input for this deposit. This entrance could be related to the West entry (Penal infilling), or more likely it comes from a secondary sub-vertical entry near to “Torreón”, perhaps related to the subsequent TD8 deposits^29^. This layer has a good collection of fossil remains, including hominins.

TD6.2.Pep is a sandy silty layer of floodplain facies (Facies E) although no channel facies is related to it (Fig. 3). Also, pH and carbonate analyses reveal that TD6.Pep has the lowest pH and carbonate content of TD6 (Fig. 6). This is indicative of alteration of the layer.

TD6.1 has a similar evolution to that of TD6.2. TD6.1.4 is a sediment gravity flow from the north-west direction that was present in the lower layers, as in TD6.2.1. Then, fluvial flows were reactivated in Gran Dolina. TD6.1.3, TD6.1.2 and TD6.1.1 were deposited by water flows. The positions of these fluvial layers are the same as in TD6.2.1, TD6.2.2 and TD6.2.3 proving that the fluvial flow had the same course (Fig. 3). The three fluvial layers of TD6.1 have associated floodplain facies (Facies E) that are different from the floodplain facies described in TD6.2 due to the lack of clasts and the low quantity of archaeological remains (Table 3), and the micromorphological data^25^. This means that the second entry in the south of Gran Dolina was not yet active, and Facies E had insufficient energy to carry the remains.

**Figure 5:**
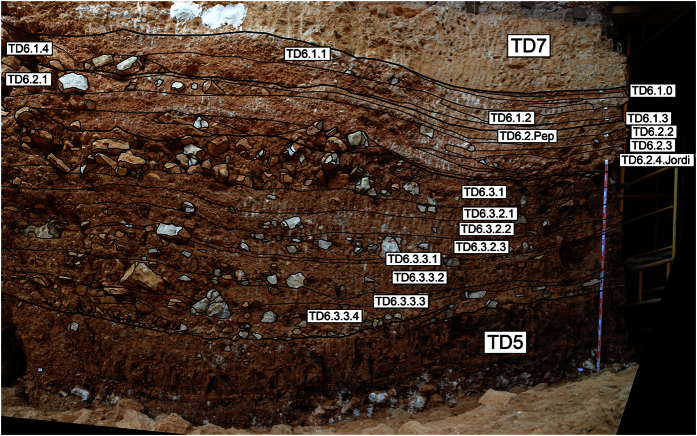
Section of TD6 showing the boundaries of the main stratigraphic layers.

**Figure 6:**
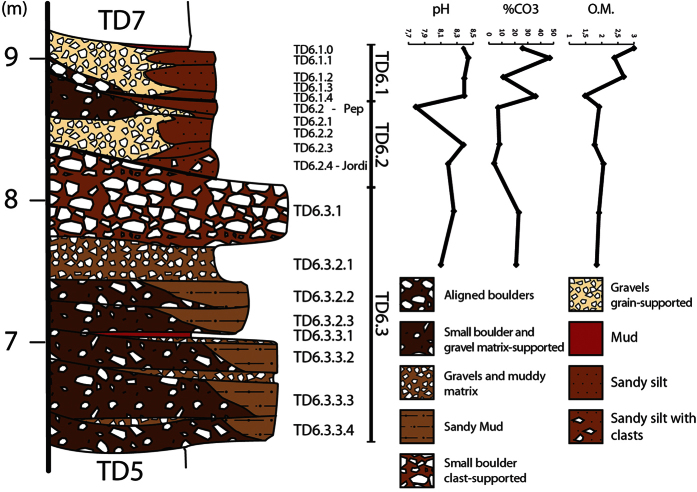
Stratigraphic and sedimentary column of the TD6 unit of the Gran Dolina site and the chemical data. Carbonates and organic matter are displayed as percentages.

Finally, TD6 finishes with a hyena coprolite layer and a red decantation layer (Facies G) indicating a slow sedimentation rate (Figs 5 and 6). Both indicate a moment of stability inside the cave at the end of TD6, and a major temporal hiatus between TD6 and TD7.

### *H. antecessor* – outside or inside the cave?

**Figure 7:**
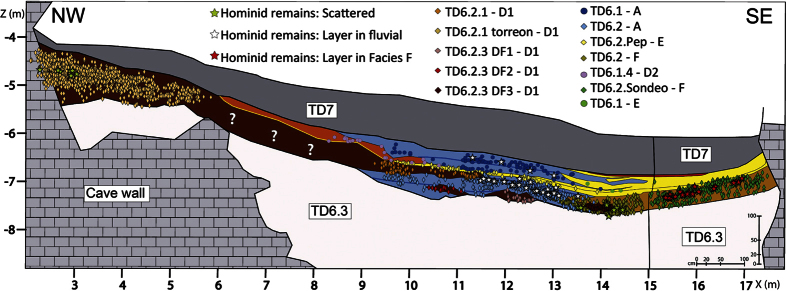
Spatial distribution of the fossils in TD6.1 and TD6.2 sub-unit section in the Gran Dolina site. The remains have been separated by sedimentary facies. Only remains within 50 cm of the section have been plotted to avoid lateral facies changes. Question marks indicate no excavated area. The projections were done by Arcgis 10.2 software.

The hominin fossils in the TD6 unit occur in sedimentary Facies A, D1 and F (Table 1, Fig. 7). The spatial distribution of these remains in the sedimentary facies allows us to distinguish three groups: a group of scattered fossils, a second group with layers of fossils in fluvial facies, and a third group of a layer of fossils in Facies F (Fig. 7).

In the first group, scattered hominin fossils appear in Facies D1 of layer TD6.2.2 “Torreón” (Fig. 9) in the northwest (Fig. 1), and in Facies F of layers TD6.2.2 and TD6.2.3 (Fig. 7). TD6.2.2 “Torreón” layer is a debris flow deposit from the outside, introduced into the cave by a sub-vertical entry, whereas TD6.2.2 and TD6.2.3 are sedimentary deposits of floodplain and debris flow processes. Quantitative data about the orientation and dip of the fossils are not available so as to be able to compare the archaeological and sedimentological fabrics^43^,^44^,^45^. Nevertheless, qualitative data recorded during the excavation suggest that the hominin fossils and the other fossils are found with no preferred orientation. The preferred tilt is sub-horizontal in Facies F, while a greater number of vertical fossils were registered in TD6.2.2 “Torreón” than in the other layers. In both cases, the tilts of the fossils are similar to the tilts of the clasts. These remains are included inside the sedimentary matrix (in the same way as sedimentary particles), and are not related to any stratigraphic contact or sedimentary palaeosurface. Besides, no processes have been observed in the sediments able to produce vertical migration of clasts or archaeological remains. In Facies F, the scattered hominin fossils were spread throughout the layers. But in Facies D1 of layer TD6.2.2 “Torreón”, the hominin fossils only appeared in the north-west and were close together (Fig. 7). This accumulation could indicate a stratigraphic limit that is not observable today, although there are only seven remains, which are insufficient for a spatial study, and their features are similar to the other fossil remains, which show a scatter and extent similar to the clasts. The latter suggests that this debris flow input could drag the bones. Even the mean size of the fossil assemblage is larger than the size observed in the fossil assemblage of other sedimentary facies (Table 3), indicating that there is a relationship between the sedimentary environment and the remains. *A priori*, these sedimentological features seem to suggest that the hominin fossils, and the other archaeological remains, were dragged by debris flow processes from the outside, and introduced into Gran Dolina cave.

In the second group (layers of fossils in fluvial facies), hominin fossils of Facies A are found in layers TD6.2.2, TD6.2.1 and TD6.1.2 (Fig. 7). Facies A is formed by a channel flow that dragged limestone gravels to the interior of the cave. The hominin fossils of this facies appear to be associated with lag layers of the fluvial flows, i.e. in layers of maximum energy and reactivation of the flow. The fossils are included in the gravels, and their size is within the range of the gravel (Table 3). From a sedimentological point of view, the channel flow was sufficient to drag the fossil remains, including hominins.

**Figure 8:**
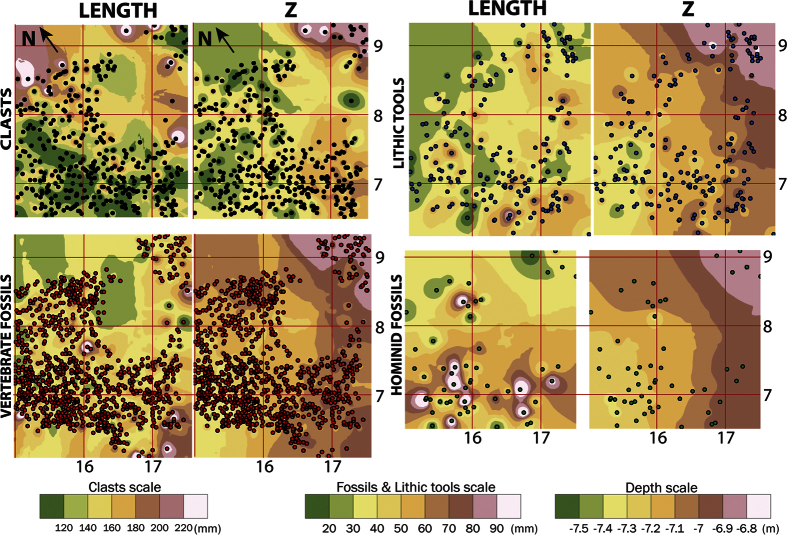
Spatial distribution and IDW (inverse distance weighting) surface of the length and depth (Z) of the test pit using only TD6.2 remains. The clasts have a separate length scale as only clasts longer than 10 cm were collected and measured. The figure was created by Arcgis 10.2 software.

The third group of hominin accumulation is characterized by the spatial distribution of the remains which form a layer inside Facies F (Figs 7 and 8). This group has the main hominin accumulation, and it occurs in TD6.2, in the test pit area, situated in the south-east (Fig. 1). In the two previous groups, the hominin remains seem to come from the outside, but this ensemble has been proposed to represent a possible home base^1^,^46^,^47^,^48^. This group has been included with others faunal remains in archaeo-stratigraphic study^35^. In the present work, Facies F has been explained as the result of two geologic processes: a floodplain environment and a debris flow input. These processes came from two different entrances: the first one related to a main entrance located at the north-west of the cave, and the second one coming from a secondary entry located to the south. The floodplain environment does not have sufficient energy to carry limestone clasts or fossil remains; this is clear when we compare it with the floodplain environment in TD6.1, where no fossils or limestones are found (Fig. 7). In addition, the spatial distribution and Z-surface of the clasts in TD6.2 show a depositional surface, describing a dip towards the west, opposite to the fluvial facies (Fig. 8). At this point, we must be careful with this interpretation because this dip was increased by subsequent post-depositional deformation. These traits indicate that the clasts were deposited from a secondary south entry, related to the debris flow process of Facies F. If the sedimentary origin of the clasts is clear, the palaeo-archaeological remains could also be introduced by geological processes.

The fossil remains and lithic tools have higher size values in Facies F, similar to Facies D1 and Facies D2 size values (Table 3), and thus also similar to TD6.2.2 “Torreón” size values. These facies are debris flow deposits, which also tend to show the same spatial distribution of archaeological remains. The latter is also observed in the hominin layer inside Facies F, in the test pit (Fig. 8). This spatial and size relationship between clasts and archaeological remains according to facies, indicates that archaeological remains could suffer the same sedimentary process as clasts, being dragged from the outside by debris flow processes. In this assumption, the hominin activity would have developed outside the cave, but very close to it, and later a geological process dragged the remains into the cave, as occurred with the hominin remains of the scattered group and the fluvial group.

The lack of selection by shape, size or skeletal elements, in addition to the lack of preferred orientation of the palaeo-archaeological remains, have been used to argue that the Aurora Stratum assemblage was not transported by any geological process^46^,^47^. But this behaviour is expected in high-density flows such as debris flows^34^,^49^,^50^,^51^. Studies about fossil preservation by debris flows describe a general horizontal or sub-horizontal tilt and no preferential orientation of elongate bones^50^,^52^,^53^. This is observed in Facies D1 and Facies F in TD6, although TD6.2.2 “Torreón” shows some remains with vertical tilt (about 10% of the remains). The latter is expected in a proximal area from a sub-vertical input.

Open-air weathering, root-marks and surface soil erosion have also been reported in the TD6.2 fossil assemblage^8^,^47^. Although these taphonomic modifications were explained initially as a direct connection of the cave to the outside, they also support an outside origin for the hominin and fossil assemblage. These sub-aerial weathering features were only found in 5.6% of the remains^8^, suggesting a short time exposed^47^.

Carbonell *et al*.^1^ commented that all phases of the reduction sequence of the exploited raw material are represented in the test pit of TD6. In addition, two sets of refitted artefacts were found. This archaeological evidence suggests that the tool manufacturing processes were carried out inside the cave or that the archaeological assemblage did not suffer sufficient transport to disperse the archaeological assemblage. The latter possibility would be supported by an outside origin and the subsequent input to the cave by debris flow processes. Debris flows have a restricted source of sediments, moving short distances^51^ and they have a very reduced capacity to sort or disperse the transported material. Landscape reconstruction of the Sierra de Atapuerca indicates that the source area of sediment for Gran Dolina was very restrictive^10^, being circumscribed to the immediate slope above the cave or the adjacent cave entry. Out of this restrictive source area, the sediments were dragged to the Arlanzón River valley. The debris flow features, landscape reconstruction, accumulation of palaeo-archaeological remains and their taphonomy suggest that the debris flow processes engulfed and transported an outside hominin habitat from the Gran Dolina slope or the cave entrance, in an area with easy accessibility to the springs and wetland resources proposed for this area^10^.

Unlike the hypothesis for the origin of the archaeological remains, the discovery of the fossils is evidence of the presence of human populations in the Sierra de Atapuerca at the end of the Early Pleistocene. The first hominin layer was registered in TD6.2.4.Jordi, with hominin remains also appearing in TD6.2.3, TD6.2.2, TD6.2.1, TD6.2.Pep and TD6.1.1 layers. But TD6.2.4.Jordi is the main accumulation layer. With this in mind, it is possible that the hominin accumulations found in Facies A were eroded from the layer of TD6.2.4.Jordi, and therefore they should not indicate a hominin occupation of the Sierra de Atapuerca. For the TD6.2.2 “Torreón”, the facies position in the north-west makes it difficult to believe that the hominin fossils were eroded from Facies F. On the one hand, if we assume the outside hypothesis, the TD6.2.2 “Torreón” deposit could erode the same outside hominin accumulation where the fossils of Facies F were. On the other hand, if we assume the inside hypothesis, this hominin accumulation would indicate a stratigraphic limit inside TD6.2.2 “Torreón” and perhaps it was related to the remains of the other facies. Even so, as the outside hypothesis of TD6.2.2 “Torreón” has been discussed before, a different episode of hominin presence is the more plausible explanation. For this, the presence of *H. anteccesor* in the Sierra de Atapuerca was recorded in several sedimentary layers, indicating an interval and not a punctual presence.

If we assume the outside hypothesis, the date of the fossil remains could be older than the currently know. Although, the taphonomic features indicates a very short time to external exposition.

The vertebrate fossil faunas of TD6.1 and TD6.2 show common features from a taphonomic point of view. Both stratigraphic units are highly cemented in the excavated area because of the continuous percolation of carbonates from the limestones during all the period of deposition and formation of the site. Consequently, most of the recovered objects show adhered sediments from the facies in which they were deposited. During the excavation, the items from floodplains and debris flows were usually covered with cemented lutites, while the objects recovered inside the channels were wrapped with a layer of calcite with small gravels. In this respect, a migration of items among facies due to post-depositional movements seems to have not been occurred.

Under these accretions of sediments, the bone surfaces are usually well preserved^54^. Most of the bones and teeth show scattered blackly spots as the result of manganese oxides formed by their deposition in a reducing waterlogged environment. This phenomenon is frequently observed in soils with large concentrations of organic matter, in which the bones are completely coated with this mineral^54^,^55^. Therefore, TD6.1 and TD6.2 seem to have not content to much organic matter in the moment of the deposition.

Some bones, mainly those located at TD6.2 “Torreon”, show some fissures and exfoliation of their surfaces, which can be related to the slight and moderate weathering degrees defined by Behrensmeyer^56^. One of the best examples is the humerus of a young individual figured in Bermúdez de Castro *et al*.^30^.

Another general feature of bone fragments from both assemblages is the angularity of their edges; nevertheless here we have to specify, since this trait is virtually absent on the items recovered inside the channel facies. In such cases, the bone edges are slightly damaged in form of rounding and polishing. These bone modifications represent ~6% of the assemblage, suggesting short movements before the sedimentation or inside the sediments. In addition, evidence of trampling in the form of random striations (~8%) is also present, reinforcing the existence of post-depositional processes related to short movements and sediment pressure.

In summary, the general taphonomic characteristics observed on the items recovered in TD6.1 and TD6.2 fit with the hypothesis presented in this work. Future detailed studies on the TD6 faunal assemblages will allow us to describe specific taphonomic features associated with each sedimentary environment.

## Methodology

The study of the stratigraphy, sedimentology and palaeo-environments of TD6 has required a detailed description of the available excavation profiles. Sediment samples have been collected from each facies in order to perform particle size analyses. Particle size sieving and laser diffraction techniques have been used. For sieving techniques, a ϕ size sieve ranging from –3 ϕ to 4 ϕ was used. Particle size has been classified following the classification of Blott and Pye^57^. Percentages of matrix and clasts were also calculated through image analysis using photogrammetry and ArcGIS 10.2 software available in CENIEH. The geochemical composition of sediments was analysed by X-ray fluorescence (PANalytical Axios) available at the Archaeometry Laboratory of the CENIEH.

A 3D survey of the stratigraphic sections and facies maps was performed using 3D laser scanning techniques (Leica C10), total stations and photogrammetry. Final figures were made with Adobe Illustrator CS5.

The archaeo-palaeontological remains were separated into four groups (clasts, lithic tools, hominin fossils and non-hominin fossils) in order to perform size, sorting and spatial analyses. The relationships between the archaeological remains and the stratigraphic units have been determined considering only the remains closest to the current stratigraphic section (maximum distance 50 cm from the section) to avoid effects produced by the dip of the layers and lateral sedimentary changes (Fig. 7). Length (longest diameter) has been used as a size indicator for the remains, since width and thickness (intermediate and shortest diameter) showed similar results. Also, the spatial distribution of the archaeological levels differentiated during the excavation has been studied through the surface interpolation of the Z-value and the item size using IDW (inverse distance weighting). These spatial analyses were carried out using ArcGIS 10.2 software.

## Conclusions

**Figure 9:**
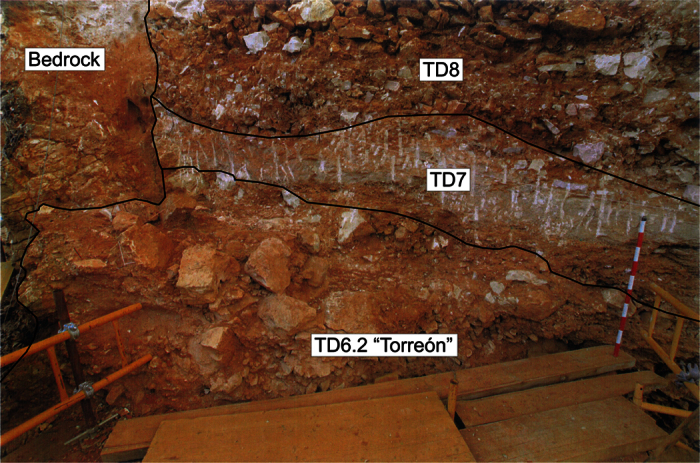
TD6.2.Torréon section located in the north-west of the Gran Dolina site. It is formed by medium boulders, clast-supported, that is defined as debris flow deposit. The hominid fossils were found between the clasts in sub-vertical position.

The TD6 unit was formed during the Early Pleistocene as a result of the sediment inputs from three entrances: a west entry, corresponding to Penal karstic infilling, a sub-vertical entry from the north-west (“Torreón” site, Fig. 9) and a secondary entry from the south-east (Test pit, Fig. 1). TD6.3 sub-unit was mainly formed by gravity flow inputs from the west entry, with an ephemeral fluvial stream across the cave, whose flow was adapted to the topography. Later, the TD6.2 and TD6.1 sub-units were formed by fluvial flow in the south-east, and a gravity flow in the north-west. The deposits of these two sub-units suggest an environment with a stream crossing the cave in the middle of the cavity, adapting to the palaeo-relief, which eventually flooded the south-east of the cave. Eventually, debris flow inputs from a sub-vertical entrance to the north-west interrupted the stream.

In this work, we describe new facies in TD6 related to energy events, and whose sediments show a close relationship with the occurrence of archaeological remains. The spatial distribution of remains coincides mainly with facies including a high proportion of boulders (facies D1 and F), and in turn, fossil and stone tools show a significant degree of size sorting related to these facies. These associations indicate a relationship between the accumulation of the remains and the sedimentation of these facies.

Previously, the palaeo-archaeological remains of TD6 have been studied extensively and their accumulation had been explained as a camp site inside the cave. Nevertheless, the relationships observed in this work seem to indicate that the camp site interpretation could be incorrect. In turn, the palaeo-archaeological accumulations found in TD6 could be explained by geological processes. The remains could have been dragged and transported by sedimentological processes to the inside of the cave from the adjacent slope or cave entry. This transport must be rapid and over very short distances since taphonomic preservation of bones is excellent, indicating a relatively short period of exposure to weather before being deposited and buried by geological agents. These accumulations are congruent with the dynamic of TD6 debris flow and the palaeogeographical reconstructions, suggesting that human activity could occur just outside the cave just before the sedimentary events.

Future detailed studies on the TD6 faunal and lithic tool assemblages using the proposed stratigraphy of this work will allow us to test the hypothesis about the source of the hominin remains and improve our knowledge of the TD6 unit.

## Additional Information

**How to cite this article**: Campaña, I. *et al*. New interpretation of the Gran Dolina-TD6 bearing *Homo antecessor* deposits through sedimentological analysis. *Sci. Rep.* **6**, 34799; doi: 10.1038/srep34799 (2016).

## Supplementary Material

Supplementary Information

## Figures and Tables

**Table 1:** Concise description of the sedimentary facies observed in TD6 unit.

Facies	Sedimentary process	Description
A	Channel	Grain-supported and grain-size decreasing gravels
B	Channel	Gravels with muddy matrix
C	Debris flow	Clast-supported medium and small boulders in close contact with muddy matrix
D1	Debris flow	Matrix-supported boulders and gravels with muddy matrix
D2	Debris flow	Aligned small boulders with gravel and muddy matrix
E	Floodplain	Sandy silt
F	Floodplain/debris flow	Sandy silt with small boulders and gravels
G	Decantation	Red mud

Particle size scale is according to Blott and Pye^57^.

**Table 2:** Sedimentary facies distribution and description of the TD6 sub-units and layers.

Sub-unit	Layers		Facies	Description
TD6.1	TD6.1.0		G	Two sub-layers are found. The bottom layer is mud with hyena coprolites and the top layer is only red mud.
	TD6.1.1		A, E	Grain-supported gravels in the middle of the section that change laterally to mud layers towards the south-east. Hominin fossils found.
	TD6.1.2		A, E	Grain-supported gravels in the middle of the section that change laterally to mud layers towards the south-east.
	TD6.1.3		A, E	Grain-supported gravels in the middle of the section that change laterally to mud layers towards the south-east.
	TD6.1.4		D2	Aligned small boulders and gravels in the middle of the section, dipping towards the south.
TD6.2	TD6.2.Pep		E	Sand and silt layer in the centre and south-east section.
	TD6.2.1		A, F	Grain-supported gravels in the middle of the section that change laterally to mud layers towards the south-east. Hominin fossils found.
	TD6.2.2		A, D1, F	Boulders and gravels with mud matrix in the north-west. Grain-supported gravels in the middle of the section that change laterally to mud layers towards the south-east. Hominin fossils found.
	TD6.2.3		A, D1, F	Grain-supported gravels in the middle of the section that change laterally to mud layers towards the south-east. Three small lenticular-shaped small boulder layers were identified. Hominin fossils found.
	TD6.2.4.Jordi		A, F, G	Mud with small boulders and gravels. It is limited by two thin red mud layers and is thinner towards the north-west. Hominin fossils found.
TD6.3	TD6.3.1		C, H	Clast-supported medium and small boulders in the centre of the section. Only matrix occurrence towards the south-east.
	TD6.3.2	TD6.3.2.1	B	Gravels with muddy matrix.
		TD6.3.2.2	D1, H	Boulders with muddy matrix, which changes laterally to only matrix towards the south-east.
		TD6.3.2.3	D1, H	Boulders with muddy matrix, which changes laterally to only matrix towards the south-east.
	TD6.3.3	TD6.3.3.1	G	5 cm of red mud.
		TD6.3.3.2	D1, B, H	Alternation of boulders and gravels with muddy matrix. Only matrix occurrence towards the south-east.
		TD6.3.3.3	D1, B, H	Alternation of boulders and gravels with muddy matrix. Only matrix occurrence towards the south-east.
		TD6.3.3.4	D1, B, H	Alternation of boulders and gravels with muddy matrix. Only matrix occurrence towards the south-east.

See Supplementary Information 1 for more details.

**Table 3:** Means and standard deviations of the length of the palaeo-archaeological remains and clasts.

Layer	Facies	Clasts			Fossils			Lithic tools		
		n	Mean	SD	n	Mean	SD	n	Mean	SD
TD6.1	A	9	128.8	23.6	84	37.3	21	12	30.2	13.4
TD6.2	A	30	157.8	58.5	296	33.5	22.8	23	36.1	19.3
TD6.2.3	A	—	—	—	24	35	16	—	—	—
TD6.2.2	D1	37	171.9	58.1	85	40.3	24.5	—	—	—
TD6.2.2 (“Torreón”)	D1	99	188.2	74.3	1506	40.5	24.6	144	33.8	18.3
TD6.2.3 (Debris flow 1)	D1	12	155	26.1	22	26.5	12	—	—	—
TD6.2.3 (Debris flow 2)	D1	13	166.1	54.7	15	42	23.9	—	—	—
TD6.2.3 (Debris flow 3)	D1	—	—	—	64	42.2	22.8	—	—	—
TD6.1.4	D2	—	—	—	19	39.2	22.7	8	27.7	11.8
TD6.2.Pep	E	17	139.7	54.7	65	33.9	19.3	7	43.5	32.3
TD6.2	F	21	143.3	74.7	305	39.3	28	19	45.2	32.4
TD6.2 (“Test pit”)	F	399	133.6	52.9	1039	41.6	28.2	196	37.5	20.5

Only remains located less than 50 cm from the section of TD6.1 and TD6.2 have been used. Values expressed in millimetres (mm).
