# Supplementary material for: New interpretation of the Gran Dolina-TD6 bearing Homo antecessor deposits through sedimentological analysis
Source: Sci Rep. 2016 Oct 7;6:34799. doi: 10.1038/srep34799 (PMC5054435; doi:10.1038/srep34799)
Supplement: Supplementary Information [file srep34799-s1.doc]

**New interpretation of the Gran Dolina-TD6 bearing *Homo antecessor* deposits through sedimentological analysis. Supplementary information.**

*I. Campaña *(1), A. Pérez-González (1), A. Benito-Calvo (1)(2), J. Rosell (3), R. Blasco (1), J.M. Bermúdez de Castro (1)(4), E. Carbonell (3)(5), J.L. Arsuaga (6)(7).*

(1) Centro Nacional de Investigación sobre la Evolución Humana (CENIEH), Paseo de la Sierra de Atapuerca 3, 09002 Burgos, Spain.

(2) Grupo Espeleológico Edelweiss, Paseo del Espolón s/n, 09071 Burgos, Spain.

(3) IPHES, Institut Català de Paleoecología Humana i Evolució Social. C/ Marcelli Domingo s/n Campus Sescelades URV (Edifici W3). 43007 Tarragona,Spain.

(4) Department of Anthropology, University College of London. 14 Taviton Street, London WC1H 0BW, UK.

(5) Universitat Rovira i Virgili (URV), Campus Catalunya, Avinguda de Catalunya 35, 43002 Tarragona, Spain.

(6) Centro de Investigación Sobre la Evolución y Comportamiento Humanos, Universidad Complutense de Madrid-Instituto de Salud Carlos III, Madrid, Spain

(7) Departamento de Paleontología, Facultad de Ciencias Geológicas, Universidad Complutense de Madrid, Madrid, Spain

*Correspondence to isidoro.campanna@cenieh.es

**Supplementary information 1**

**Sedimentary facies**

**Facies A: Grain-supported** **channel**

This facies is formed by grain-supported and normal graded gravels with a low quantity of matrix, and including trough cross-bedding. Gravels occupy about 60% by weight and they are well sorted, subangular limestone gravels about 3–5 cm in size. The sedimentary layer adopts a lenticular shape and is composed of several aggradation phases where clastic lags appear at the base of each phase. We noticed at least three lags in TD6.1 and another three lags in TD6.2. The particle size, well-sorted nature and lenticular-shape suggest a fluvial flow process forming this facies, more specifically, a channel facies ^45^. Lag layers are formed by reactivation of fluvial flow after channel migration. Facies A is located in the central position of the section, where it overlies debris flow facies, and it also lies to the north-west of Facies E (TD6.1) and Facies F (TD6.2).

**Facies B: Channel with muddy matrix**

This facies consists of gravels supported by a muddy matrix or in loose contact. The matrix is composed of sand and mud and this represents about 60% of the total weight. The gravels are composed of sub-angular limestone and they are unsorted. Facies B forms lenticular-shaped layers <25–30 cm thick and 1 m in length. Its characteristics suggest that Facies B was formed by fluvial flows. This facies occurs in TD6.3 and it is present above Facies D1.

**Facies C: Clast-supported** **debris flow**

Facies C is formed by clast-supported medium and small boulders in close contact with a muddy matrix. Clasts in the section represent about 40% of the facies and are medium to small subangular elongate boulders. The matrix is mud with a low content of gravel and sand. This facies appears as a layer in the centre of the section of TD6.3 and is up to 80 cm thick. The localization of the maximum thickness in the centre of the section and the shape of the layer suggest a different direction of sediment input from Facies D1 and Facies D2. This facies is unsorted and with a massive amount of sediment of different sizes that assigns it to debris flow facies ^46^.

**Facies D1: Matrix-supported** **debris flow**

This facies is characterized by a mixture of chaotic and unsorted sediments from medium sized boulders to gravels, sands and mud. Facies D1 is mainly composed of matrix-supported boulders and gravels with a muddy matrix, including local clast-supported areas. The clasts in this facies represent 30–35% of the area measured in the stratigraphic section. The matrix is mainly composed of about 75% of clayey silt with about 25% of gravel. The amount and size of boulders decreased towards the south-east, towards the dip direction of this facies. These characteristics indicate that the entrance of this facies was positioned in the north-west. Facies D1 is found in TD6.2 and TD6.3.

**Facies D2: Aligned-clasts debris flow**

Facies D2 is formed by clast-supported small boulders with a gravel and muddy matrix. It is characterized by aligned clasts that indicate the entrance direction and could suggest a unique punctual sediment input for its formation. This facies occurs in TD6.1, with a constant thickness of about 0.3 m. The layer containing this facies dips towards the south-east, suggesting a north-west location of the entrance. Boulders are sub-angular and tabular limestone ranging from 10 to 30 cm in size, and they represent about 40% of the section area. The matrix has up to 25% gravel. Since this facies is similar to Facies D1, we have also related this facies to a debris flow process.

**Facies E: Floodplain**

Facies E is yellowish red massive coarse and fine silt, including about 10–0% gravel, 25–15% sand and 80–65% mud. This facies appears associated with Facies D, which is situated to the north of Facies E. Due to its traits, particle size and association with Facies D, this facies is interpreted to be a floodplain deposit. It was formed by overbank events related to channel facies. This facies always appears in the south of the section in TD6.1.

**Facies F: Floodplain and debris flow**

Facies F is composed of silts and clays with small boulders and gravel. It is similar to Facies E, showing the same matrix particle size distribution and the same massive structure. As with Facies E, Facies F is associated with Facies A, but Facies F is only found in TD6.2 (Fig. 3). Because of these traits, we interpret Facies F as also being floodplain facies. Nevertheless, this facies includes several limestone clasts, which are medium to very small sub-angular boulders that represent about 20% of the section. These boulders appear dispersed in the layers as matrix-supported, unsorted, structureless and with no preferred orientation. The presence of these clasts does not agree with a floodplain environment as observed in Facies E, and seems to indicate a secondary sedimentological process in the development of Facies F. The amount, size and shape of the limestone boulders suggest a gravity flow process and the dip of the layers towards the north-west (Fig. 4) indicates a southern entry, although this dip is exaggerated by the deformation process. Facies F is explained as the result of two geological processes: floodplain and debris flow. Because of the similar matrix characteristics produced by these two processes, the lateral change separating both environments could not be mapped.

**Facies G: Decantation**

Facies G is composed of thin (<10 cm) yellowish red clays. It contains <20% sand, and no gravels are present. Facies G is recognizable by its homogeneity and clay content. According to these characteristics, we have interpreted this facies as sediment transported into the cave by suspension and deposited by decantation. This facies appears at TD6.3.3, TD6.2 and at the top of the TD6 unit (TD6.1.0), in the south-east of the section.

**Facies H: Mud flow**

This facies is composed of yellowish red silt and clay with sparse limestone clasts. It is 5 to 30 cm thick and forms a tabular layer of unsorted and massive fine sediments. Particle size is about 20% sand, varying from fine to very fine grain size, and 80% mud, more silty in the proximal area and more clayey towards the distal area. Limestone clasts are small boulders and gravels that are poorly represented (<15% of the area measured in the stratigraphic section), and they usually occur in the proximal area. This facies is frequently found in the south-east of the section of TD6.3, and is related to distal sediments of Facies D1 and Facies C, where a progressive lateral facies change has occurred. This facies is interpreted as mud flow facies.

**Supplementary information 2**

**Geochemical element analysis**

|  | TD6.3.3.4 | TD6.3.3.1 | TD6.3.1 | TD6.2.4 | TD6.3.pep | TD6.1.3 |
| --- | --- | --- | --- | --- | --- | --- |
| SiO_2_ | 47.09 | 40.9 | 52.71 | 44.05 | 40.85 | 34.94 |
| Al_2_O_3_ | 8.59 | 9.29 | 11.31 | 9.02 | 4.73 | 6.5 |
| Fe_2_O_3t_ | 3.23 | 3.35 | 4.21 | 3.34 | 1.66 | 2.36 |
| MnO | 0.06 | 0.06 | 0.07 | 0.07 | 0.04 | 0.05 |
| MgO | 0.57 | 0.6 | 0.66 | 0.53 | 0.29 | 0.4 |
| CaO | 19.14 | 22.43 | 13.18 | 20.76 | 27.22 | 28.9 |
| Na_2_O | 0.12 | 0.1 | 0.13 | 0.12 | 0.11 | 0.1 |
| K_2_O | 1.51 | 1.45 | 1.83 | 1.42 | 0.94 | 1.05 |
| TiO_2_ | 0.71 | 0.69 | 0.87 | 0.72 | 0.53 | 0.56 |
| P_2_O_5_ | 3.39 | 0.31 | 3.41 | 3.74 | 3.6 | 2.24 |
| LOI | 15.58 | 20.83 | 11.6 | 16.22 | 20.01 | 22.87 |

Table SI2. Elemental composition of six layers of TD6. Values are displayed as percentages.

The geochemical data do not show important changes. The main elements present in the layers of TD6 are SiO_2_ and CaO. The first is from terra rossa source, rich in this element. The last came from the limestone of the cave, both fragments of the rock and secondary precipitation. The rest of elements have minor variations that could due to the texture of the sediment and post-depositional process. Anyway, these little variations of the geochemical data are not related to the sedimentary facies.
